# The miRNA transcriptome of cerebrospinal fluid in preterm infants reveals the signaling pathways that promote reactive gliosis following cerebral hemorrhage

**DOI:** 10.3389/fnmol.2023.1211373

**Published:** 2023-09-18

**Authors:** Andriana Gialeli, Robert Spaull, Torsten Plösch, James Uney, Oscar Cordero Llana, Axel Heep

**Affiliations:** ^1^School of Medicine and Health Science, Research Centre Neurosensory Science, University of Oldenburg, Oldenburg, Germany; ^2^Bristol Medical School, Translational Health Sciences, Dorothy Hodgkin Building, University of Bristol, Bristol, United Kingdom

**Keywords:** cerebrospinal fluid, miRNA, gene ontology, intraventricular hemorrhage, neural progenitor cells, JAK/STAT pathway, astrogliosis

## Abstract

**Introduction:**

Germinal Matrix-Intraventricular Haemorrhage (GM-IVH) is one of the most common neurological complications in preterm infants, which can lead to accumulation of cerebrospinal fluid (CSF) and is a major cause of severe neurodevelopmental impairment in preterm infants. However, the pathophysiological mechanisms triggered by GM-IVH are poorly understood. Analyzing the CSF that accumulates following IVH may allow the molecular signaling and intracellular communication that contributes to pathogenesis to be elucidated. Growing evidence suggests that miRs, due to their key role in gene expression, have a significant utility as new therapeutics and biomarkers.

**Methods:**

The levels of 2,083 microRNAs (miRs) in 15 CSF samples from 10 infants with IVH were measured using miRNA whole transcriptome sequencing. Gene ontology (GO) and miR family analysis were used to uncover dysregulated signalling which were then validated in vitro in human foetal neural progenitor cells treated with IVH-CSF.

**Results:**

Five hundred eighty-seven miRs were differentially expressed in the CSF extracted at least 2 months after injury, compared to CSF extracted within the first month of injury. GO uncovered key pathways targeted by differentially expressed miRs including the MAPK cascade and the JAK/STAT pathway. Astrogliosis is known to occur in preterm infants, and we hypothesized that this could be due to abnormal CSF-miR signaling resulting in dysregulation of the JAK/STAT pathway – a key controller of astrocyte differentiation. We then confirmed that treatment with IVH-CSF promotes astrocyte differentiation from human fetal NPCs and that this effect could be prevented by JAK/STAT inhibition. Taken together, our results provide novel insights into the CSF/NPCs crosstalk following perinatal brain injury and reveal novel targets to improve neurodevelopmental outcomes in preterm infants.

## 1. Introduction

Improvements in neonatal care have led to an increase in the survival of very low birth weight premature infants. Nevertheless, the most serious complication following premature birth is the development of periventricular germinal matrix hemorrhage (GMH) secondarily extending into the ventricles of the brain (intraventricular hemorrhage; IVH; Ballabh, 2010). Twenty-five percent of very low birth weight preterm infants (birth weight < 1,000 g, <32 weeks gestational age) (Ballabh, 2010) will develop GMH-IVH (Inder et al., 2018) and 40–60% of infants with extended IVH develop progressive post-hemorrhagic ventricular dilatation (PHVD) with considerable mortality and morbidity in surviving infants (Limbrick et al., 2010; Robinson, 2012). Preclinical and clinical data in preterm infants developing PHVD indicates that the neuropathology following GMH and IVH not only disrupts the ventricular lining but also the underlying ventricular and subventricular zones – key neurogenic niches – while biochemically, GMH-IVH presents with upregulation of pro-inflammatory cytokines, hemoglobin degradation and neurotrophic factor dysregulation in the cerebrospinal fluid (CSF) (McAllister et al., 2017).

Progressive PHVD, in extreme preterm infants, is typically managed by CSF removal through ventricular access devices to control ventricular dilatation (McCrea and Ment, 2008; de Vries et al., 2019). This drained CSF represents a unique opportunity to characterize ongoing pathophysiological mechanisms following IVH. We have previously shown that miRNA (miR)-containing extracellular vesicles populate the CSF in preterm infants with PHVD (Spaull et al., 2019) and increased levels of pro-inflammatory miRs have been reported in IVH-CSF samples (Fejes et al., 2020).

miRs are small non-coding RNAs, that regulate post-transcriptional gene expression in a sequence-specific manner (Bartel, 2004; He and Hannon, 2004). Multiple studies have shown their abundance in the central nervous system (CNS) and their importance in brain development (Cho et al., 2019; Ponnusamy and Yip, 2019). miRs can be loaded in 30–5,000 nm small lipid particles, called extracellular vehicles (EVs), which are released by almost all cell types into the extracellular space and biofluids and can play a role in trans-cellular communication (Théry et al., 2006; Surgucheva et al., 2012). Dramatic changes in miR expression in biofluids from patients with neurological diseases, e.g., traumatic brain injury (Bhomia et al., 2016; Martinez and Peplow, 2017), multiple sclerosis (Munõz-San Martín et al., 2019; Perdaens et al., 2020), Alzheimer’s (Dangla-Valls et al., 2017; Cha et al., 2019), and Parkinson’s (Marques et al., 2017; dos Santos et al., 2018), have been previously reported.

We therefore speculated that disruption of miR regulation following GMH-IVH in preterm infants might directly impact neural stem/precursor pools in the periventricular germinal matrix thereby disrupting pivotal processes of brain development. This study aimed to identify changes in the miR transcriptome in the CSF of infants with progressive GMH-IVH and to investigate how miR dysregulation contributes to the pathophysiology.

## 2. Materials and methods

### 2.1. CSF sample collection

CSF samples were collected from 10 preterm infants born between 23 and 37 weeks of gestation (birth weight < 1,000 g) and treated for GMH-IVH at the regional tertiary Neonatal Intensive Care Unit at Southmead Hospital in Bristol, United Kingdom. Patients with suspected CNS infection (defined as raised white cell count or positive bacterial culture in the CSF), known genetic abnormalities, or presenting with CNS malformation were excluded from the study. IVH was diagnosed as part of clinical assessment with serial trans-fontanel ultrasound and quantified according to the Levene and Starte (1981). The indication for external CSF drainage was standardized following the ELVIS study protocol (de Vries et al., 2019). External CSF drainage was indicated by ultrasound measurement of ventricular index (VI) and anterior horn width (AHW) exceeding 97th percentile for gestational age, initially undertaken via lumbar puncture before insertion of a ventricular access device (VAD) if ventricular enlargement persisted above the 97th percentile (Department of Paediatric Neurosurgery, Bristol Royal Hospital for Children, Bristol, United Kingdom).

CSF used in this study was the excess fluid remaining after routine testing of samples taken for clinical indications following informed parental consent. Initial sample volume was 10 mL/kg body weight, the mean leftover CSF volume available for testing was 4.4 mL. Ethical approval for analysis of the CSF was obtained from NHS Research Ethics Committee (REC number: 15-YH-0251). From each patient, CSF was collected at different time points over the treatment course. CSF was centrifuged to remove cellular material and the supernatant frozen at −20°C within an hour of collection and transferred to −80°C as soon as practicable. CSF sample characteristics are indicated in Supplementary Table 1.

### 2.2. microRNA expression profiling

HTG Molecular Diagnostics miR whole transcriptome assay^1^ was used for miR profiling. 12.5 μL of CSF were processed for screening of 2,083 miRs by next generation sequencing (NGS). Fifteen CSF samples along with human brain RNA control technical replicates were randomized and analyzed. Post-sequencing quality control (QC) metrics were applied to all samples. QC metrics detect three different failure modes: QC0 for degraded RNA or poor-quality sample, QC1 for insufficient read depth and QC2 for minimal expression variability. Demultiplexed FASTQ files were returned from the sequencer and aligned with the probe list. Raw, QC raw, log_2_ CPM (counts per million) and median normalized data were provided. All samples passed the quality control metrics.

### 2.3. microRNA sequencing analysis

HTG EdgeSeq Reveal software^2^ version 4 was used for analysis and visualisation of miR sequencing results. DESeq2 differential expression test, provided by HTG EdgeSeg Reveal software, was used for statistical analysis of differential expressed miRs across duration of CSF sampling after IVH.

### 2.4. Gene ontology analysis

Gene ontology analysis was performed in miRs with significant differential expression (Fold change>2, *p* < 0.05) between the early and late samples by miEAA 2.0 (Kern, 2020). Over-Representation Analysis (ORA) (FDR < 0.05, minimum required hits per sub-category: 2). Four miRs were classified as “unknown entries” and removed from the list.

### 2.5. miRNA family analysis

The visualization of the miR families among our significantly dysregulated miRs (Fold change>2, *p* < 0.05) was performed by miRViz tool (Giroux et al., 2020), using the “Seed2_7” network option. The families containing at least 3 significantly dysregulated miRs, where selected, and the potential miR-target interactions analysis and network of dysregulated miRs was performed by Mienturnet (MirTarBase; Licursi et al., 2019) (Network set-up: minimum number of miR-target interactions: 2, FDR < 0.05, filter by evidence categories: strong evidence only).

### 2.6. Cell culture

Human cortical tissue (8–10 weeks gestation) was collected following routine terminations of pregnancies at the University of Cardiff. The methods of collection conformed to arrangements recommended by the Review of the Guidance on the Research Use of Fetuses and Fetal Material (1989) and the Health Authorities Act (1995).

Human fetal neural progenitor cells (hfNPCs) were cultured as neurospheres in non-adherent culture in growth media high glucose Dulbecco’s modified Eagle’s medium (DMEM, Invitrogen), F12 (Invitrogen) with 3:1 ratio DMEM/F12, 1% penicillin–streptomycin (Sigma), 2% glutamax (Invitrogen), 2% B27 supplement, 20 ng/mL fibroblast growth factor (FGF-2, Peprotech), 20 ng/mL epidermal growth factor (EGF, Sigma), and 5 μg/mL heparin (Sigma). Half of the growth medium was replenished twice a week, and the cells were mechanically passaged with a McIlwain tissue chopper as previously described (Cordero-Llana et al., 2011). hfNPCs were cultured at 37°C and at 5% CO_2_ in a humidified incubator.

### 2.7. hfNPCs differentiation

hfNPCs were plated in 8-well NuncTM Lab-TekTM chamber slides (Invitrogen) for immunostaining or culture plates for RNA extraction. Both slides and plates were pre-coated with PDL (Sigma, United Kingdom) overnight at 4°C. PLD was then removed, slides were washed with sterile water and coated with 1 mg/mL laminin (Sigma) for 1 h in the incubator (5%CO_2_, 37°C). Excess laminin was removed, and neurospheres were plated and cultured in differentiation media: 3:1 DMEM:F12 1% penicillin–streptomycin, 1% glutamax and 1% N2 supplement. 10% of IVH-CSF were added into the plates and cells were allowed to differentiate for 7 days. JAK inhibitor I (Generon) was added at the concentration of 10 μM. Both CSF and JAK inhibitor I were added 5 h after hfNPCs were plated.

### 2.8. Immunostaining

Cells were fixed in 4% PFA for 20 min. After permeabilization (ice cold methanol at −20°C for 20 min) and blocking (10% normal goat serum in PBS, for 2 h at room temperature) cells were incubated overnight at 4°C with primary antibodies: rabbit anti-GFAP (1:1000, DAKO), mouse anti-Tuj1 (1:500, Biolegend), mouse anti-STAT3 (1:200, cell signaling), rabbit anti-phospho-STAT3 (1:200, cell signaling), rabbit anti-p44/42 MAPK (1:200, cell signaling), rabbit anti-phospho-p44/42 MAPK (1,200, cell signaling). Cells were then washed and treated for 2 h with 1:500 dilution of Alexa-secondary antibodies. Finally, cell nuclei were stained with Hoechst (5 μg/μL, Sigma) for 20 min. Cells were mounted in vectashield hardset antifade mounting medium (Vector Labs). Images were obtained with either a high-resolution camera (Leica Microsystems LTd DFC340FX) using the microscope Leica DMRB or Leica confocal laser scanning microscope or IN Cell Analyzer 2000. At least three random fields per well were taken imaged the stained cells automatedly counted using IN Cell Analyzer software or manually counted using ImageJ (in cases where complex cellular morphology could not be accurately segmented using automated software).

### 2.9. RNA extraction

Total RNA was extracted from approximately 0.7 mL CSF samples from infants with IVH, using the Zymo Research Direct-zol™ RNA Microprep kit (R2061, Cambridge Bioscience, United Kingdom) according to the manufacturer’s guidelines. Total RNA concentration (ng/μL) was measured using the NanoDrop 2000c Spectrophotometer (ThermoScientific).

### 2.10. qRT-PCR

TaqMan MicroRNA Assay (Applied Biosystems™, ThermoFisher) was used for reverse transcription (RT) and real-time PCR (qPCR). PCRs were performed according to the manufacturer’s instructions. Briefly, cDNA constructs were synthesized by using 2 ng/μL total RNA and miR-specific RT looped-primers (5X). At this step, 2fmoles/μL of the synthetic cel-miR-39 (MERK LifeScience, United Kingdom) were added in all reactions. cDNA products and sequence specific primers (20x) were combined with TaqMan^®^ Universal Master Mix II and run on a StepOnePlus™ real-time PCR system (Applied Biosystems) in triplicates. The ΔΔCT method was used for relative quantification of miR expression. miR-146a & miR-204 (top upregulated based on our NGS analysis), miR-93 & miR-106b (top downregulated based on our NGS analysis), miR-17 [downregulated in exosomes isolated from GM-IVH CSF (Spaull et al., 2019)], miR-1911 [upregulated in exosomes isolated from GM-IVH CSF (Spaull et al., 2019)] were used for validation, while cel-miR-39 was used as exogenous normalizer (Supplementary Table 3). Supplementary Table 3 shows primer sequences and IDs. For gene expression analysis, the GoTaq probe RT-qPCR system (Promega) was used for the preparation of the cDNA while power SYBR green PCR master mix kit (Applied biosystems) was used for the real time PCR (qPCR). PCRs were performed according to the manufacturer’s instructions. cDNA constructs were synthesized by using 1.5 ng/μL total RNA and random primers. cDNA products and target-gene forward and reverse primers (300 nM) were combined with Power SYBR^®^ green PCR Master Mix and ran on a StepOnePlus™ real-time PCR system (Applied Biosystems) in triplicates. ΔΔCT method was used for relative quantification of miRNA expression.

### 2.11. Statistical analysis

DESeq2 differential expression test (Love et al., 2014), provided by HTG EdgeSeg Reveal software, was used for statistical analysis of differentially expressed miRs across duration of CSF sampling after IVH. Two-way ANOVA and paired t-test provided by GraphPad Prism 6 were used for statistical analysis of qRT-PCR experiments. Graphs represent mean ± SEM, N numbers are specified in figure legends.

## 3. Results

### 3.1. Distinct CSF-miR expression clusters in preterm infants with GM-IVH

**Figure. 1:**
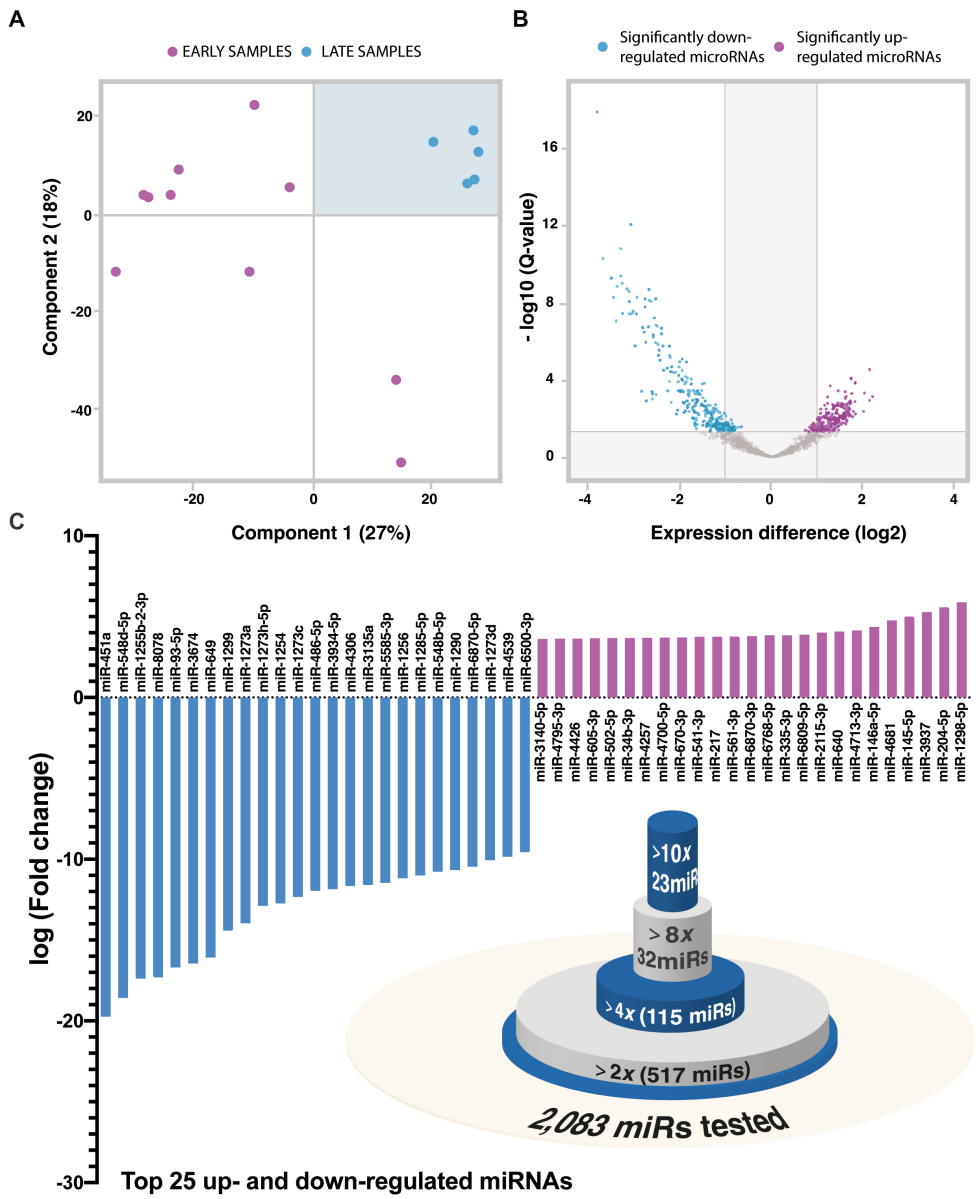
**(A)** Principal component analysis (PCA) of sequenced IVH-CSF samples. Early CSF is labeled as red while late CSF as blue. **(B)** Volcano plot of the significant differentially expressed miRNA between early and late IVH-CSF. DESeq2 differential expression test was performed. **(C)** Top 25 up- and down-regulated miRNA in the late IVH-CSF compared to early IVH-CSF. Venn-diagram of the differentially expressed miRNAs.

We performed next generation sequencing (NGS) of CSF from 10 preterm infants with GM-IVH to profile the whole miR transcriptome (Supplementary Table 1). CSF was collected at different time-points over GM-IVH management, as it is known that perinatal brain injury follows a timeline including three phases (acute, secondary and tertiary phase of injury; Fleiss and Gressens, 2012). Principal component analysis (PCA) was performed to investigate the similarity of the miR expression profiles of the different CSF samples (Figure 1A). PCA identified a clear cluster of samples (*n* = 5) within the top-right quadrant, interestingly these were all samples taken towards the resolution of the IVH incident (extracted between 60 and 135 days after IVH). We thereafter refer to this group as “late-samples”-referring to tertiary phase of brain injury. PCA also revealed a more heterogeneous cluster (n = 8) on the top left quadrant. This encompasses samples taken between 1 and 30 days after the hemorrhagic incident and we thereafter refer to this group as “early-samples.” Two samples – bottom right quadrant – did not fit either of these two groupings. Interestingly, one of these belongs to patient 1 (Figure 1A), who was later confirmed to present congenital ventriculomegaly and not GM-IVH. The other outlier corresponds to the only patient in our study that was admitted to a different recruitment site and as such we were unable to confirm their GMH-IVH diagnosis. Therefore, both samples were excluded from subsequent analysis.

### 3.2. Five hundred eighty-seven miRs are differentially expressed in the CSF of premature infants over the course of GM-IVH

To further characterize these two clusters, we looked at which miRs were differentially expressed. Out of 2,083 miRs, 587 were differentially expressed (DESeq2, *p* < 0.05, Figure 1B) with 325 down-regulated and 262 up-regulated miRs over GM-IVH management (Supplementary Data). From those miRs, 115 miRs had fold change above 4, 32 miRs had fold change above 8 and 23 miRs had fold change above 10 (Figure 1C). The top 10 down-regulated miRs were miR-451a, miR-548d-5p, miR-1255b-2-3p, miR-8,078, miR-93-5p, miR-3,674, miR-649, miR-1,299, miR-1273a, miR-1,273 h-5p, while the top-10 up-regulated miRNAs were miR-1,298-5p, miR-204-5p, miR-3,937, miR-145-5p, miR-4,681, miR-146a-5p, miR-4,713-3p, miR-640, miR-2,115-3p, miR-6,809-5p (Figure 1C). These results highlight the dramatic changes in miR content in the CSF during GM-IVH management.

To validate the NGS results, six miRs were selected and quantified by qPCR. These miRs were (A) the two top significantly up-regulated (miR-204, miR-146a), (B) the two top significantly down-regulated (miR-93, miR-106b) miRNAs with high expression in the CSF, based on our NGS analysis and (C) two miRs which we had previously reported (Spaull et al., 2019) to be downregulated (miR-17) and upregulated (miR-1911) in exosomes isolated from CSF of infants over the course of GM-IVH.

All tested miRs showed the same direction of change between qPCR and NGS (Supplementary Figure 2), and miR-93 (*p* = 0.0193) was significantly downregulated in late samples, confirming the robustness of our NGS approach. The exogenous cel-miR-39 was used for normalization of qPCR experiments.

### 3.3. Gene ontology analysis identifies key signaling pathways involved in GM-IVH progression

**Figure. 2:**
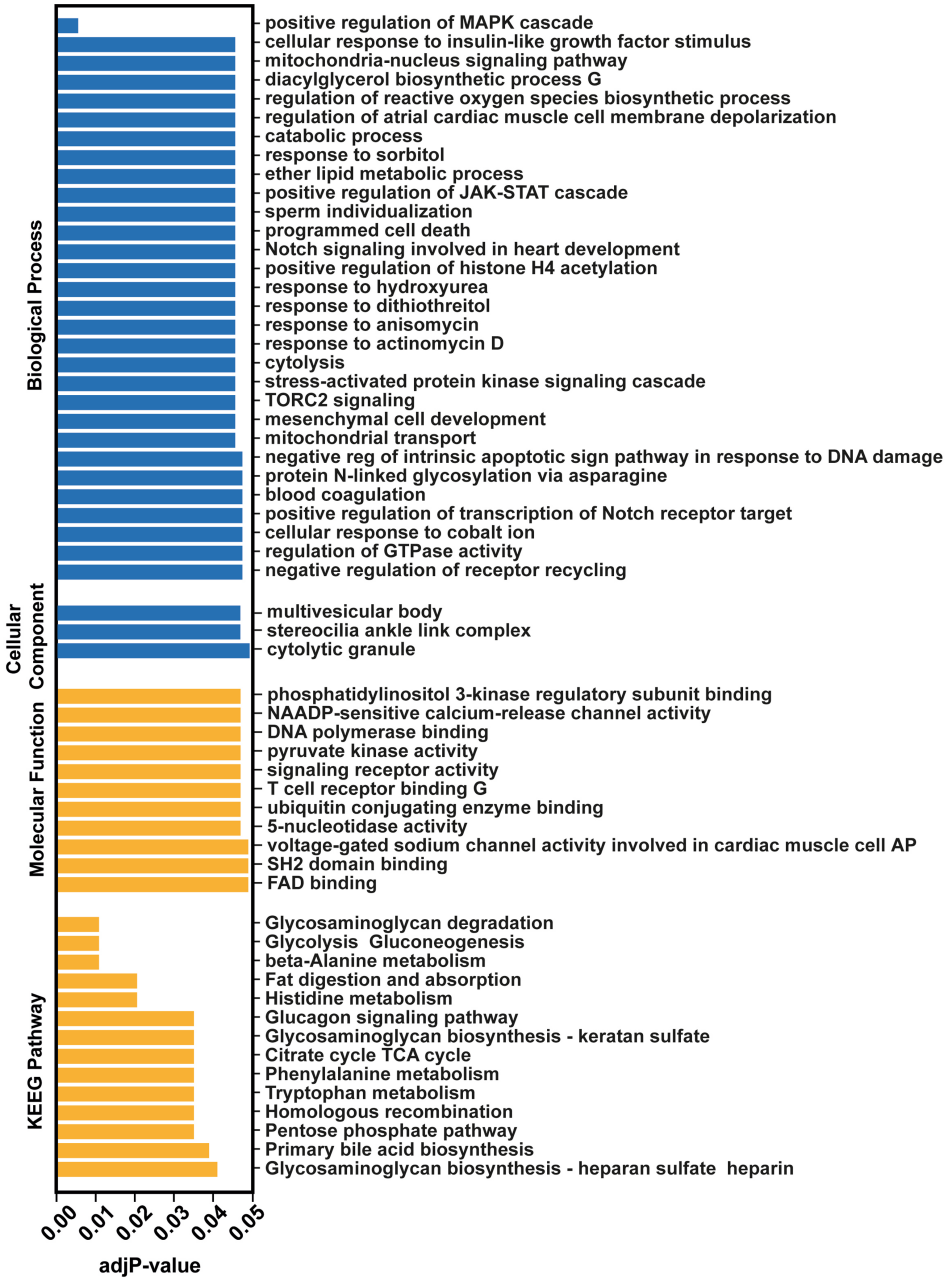
Over-Representation Analysis (ORA) of the significantly differentiated miRNAs (Fold change>2, *p* < 0.05) by miEAA 2.0 tool.

GO analysis was performed on all the significantly differentially expressed miRs (Fold change>2) between the two groups (517 miRs). This analysis revealed the positive regulation of MAPK cascade (adj *p*-value = 0.0064325), cellular response to insulin-like growth factor stimulus (adj *p*-value = 0.0472509), mitochondria-nucleus signaling pathway (adj *p*-value = 0.0472509) and positive regulation of JAK–STAT cascade (adj *p*-value = 0.0476272) as the most targeted pathways (Figure 2). This highlights important biological process that may play a role in the prognosis of GM-IVH.

### 3.4. Nine miR families are differentially expressed in the CSF of premature infants over the course of GM-IVH

**Figure. 3:**
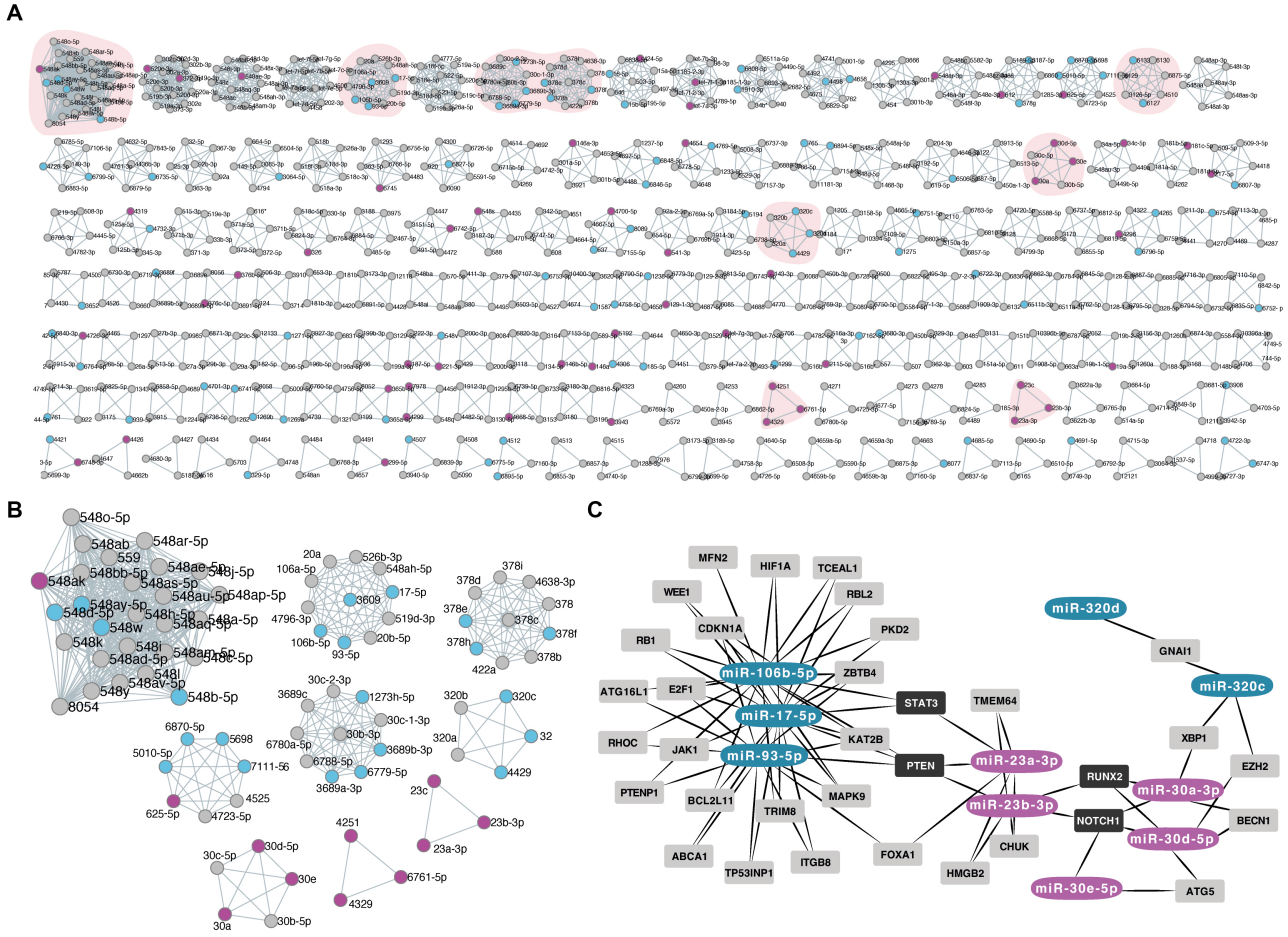
**(A,B)** Panel of the miRNA families based on their common seed sequence by miRViz tool. The down-regulated miRNAs in late IVH-CSF are depicted with blue color while the up-regulated miRNAs with magenta. The small box shows the miRNA families that had 3 or more family members from our list of deregulated miRNAs. **(C)** Diagram of the common targets of the miRNA families by Mienturnet (MirTarBase), diagram made with Cytoscape. Dark gray boxes label the common targets between miRNA families members.

We then identified miRs that share the same seed sequence (families which will have similar mRNA targets) in our dysregulated group. Nine miR families had 3 or more family members dysregulated between early and late CSF samples: miR-548 family, miR-17 family, miR-3,689 family, miR-378, miR-6,870 family, miR-320, miR-30 family, miR-4,251, and miR-23 (Figures 3A,B, Supplementary Table 4).

Then, we investigated the shared target interactions of the miRs belonging to those nine families. We filtered using only miRNA-target interactions “*validated by strong experimental methods*.” Members of the miR-17, miR-23, miR-30, and miR-320 families showed common targets and in particular STAT3 (Figure 3C, Supplementary Data).

### 3.5. IVH-CSF regulates MAPK and JAK/STAT signaling in human fetal neural progenitor cells

To further validate the predictions of our GO analysis, we focused on MAPK pathway – the most significant targeted pathway. We also selected the JAK/STAT as (A) our Mienturnet (MirTarBase) analysis revealed that members of the miR-17, miR-23, miR-30, and miR-320 families showed strong interactions with common targets including PTEN, NOTCH1, RUNX2, AND STAT3 (B) the JAK/STAT signalling pathway plays a key role in inflammation, tissue repair and apoptosis (Hu et al., 2021) and (C) Many drugs targeting this pathway, have already been approved for preclinical and clinical use (Hu et al., 2021). We hypothesized that IVH-CSF could indeed modulate MAPK and JAK/STAT signaling in target cells. For this, hfNPCs were differentiated in the presence of either early or late IVH-CSF. The expression levels of MAPK protein were not affected by IVH-CSF treatment (Supplementary Figures 3B,E), however exposure to late IVH-CSF resulted in decrease in the number of cells with detectable levels of MAPK (late mean: 0.7189, non-treated mean: 1.113, SEM:0.1118 adj-*p* = 0.0378, Supplementary Figures 3B,D) and a decrease in the levels of phosphorylated p44/42 MAPK (pMAPK+) (late mean: 0.6293, non-treated mean: 1, SEM: 0.1158, adj-*p* = 0.0416, Supplementary Figures 3C,F) compare to non-treated cells (Supplementary Figure 4).

**Figure. 4:**
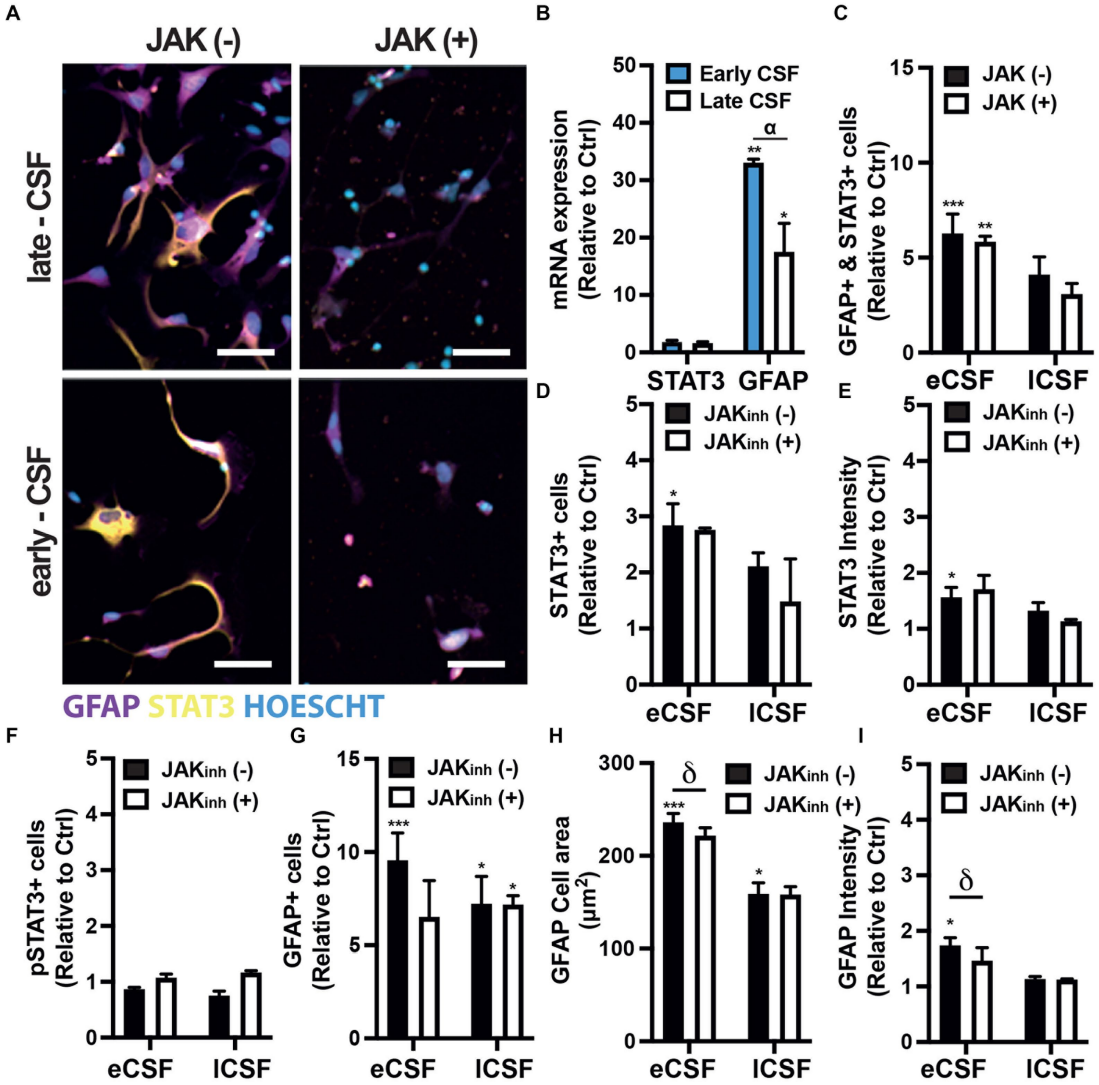
**(A)** Representative immunofluorescence images of human fetal NPCs treated with either early- or late-CSF with/without JAK inhibitor stained for GFAP and STAT3. Scale bars 50 μm. **(B)** Relative mRNA expression of STAT3 and GFAP in hfNPCs treated with either early- or late-CSF. **(C)** Relative quantification of the number of cells expressing both STAT3 and GFAP. **(D)** Quantification of the number of STAT3+ cells. **(E)** Quantification of STAT3 intensity. **(F)** Quantification of the number of pSTAT3+ cells. **(G)** Relative number of GFAP+ cells. **(H)** Relative cell area covered by GFAP+ cells. **(I)** Relative quantification of GFAP intensity. All experiments were repeated with early or late CSF samples from *n* = 3 patients (Supplementary Table 1: patients 4, 6 & 7). One-way / two-way ANOVA and Dunnett post-hoc test. Bars represent mean + SEM. (*) represents the significant difference compared to non-treated cells, (α) represents the significant differences between treatments with early and late CSF samples, (δ) represents the significant difference between JAKinh (+) and JAKinh (−).

The relative expression of *Stat3* mRNA in hfNPCs treated with early and late IVH-CSF showed a close to significant increase compared to non-treated cells (early adj-*p* = 0.0599 and late adj-*p* = 0.1440; Figure 4B). Exposure to early but not to late IVH-CSF resulted in an increase of STAT3+ cells (early mean:3.204, non-treated mean: 1, SEM: 0.6669, adj-*p* = 0.0478) compared to non-treated cells (Figures 4A,D, Supplementary Figure 4). Similarly, the expression levels of STAT3 protein were also increased after exposure to early IVH-CSF (early mean: 1.729, non-treated mean: 1, SEM: 0.1920, adj-*p* = 0.0196, Figures 4A,E) but were unchanged after exposure to late IVH-CSF. No changes in the levels of phosphorylated Tyr705 STAT3 were detectable for any of these conditions (Figure 4F, Supplementary Figures 3A, 4). Together, these results show that both JAK/STAT and MAPK pathways may play a role in modulating IVH related pathology at different time points during the conditions progression.

### 3.6. IVH-CSF promotes reactive gliosis, which is mediated by JACK/STAT3 at early stages of IVH progression

As the JAK/STAT pathway has been associated with reactive gliosis and astrocyte differentiation (Haim et al., 2015; Ceyzériat et al., 2016, 2018), we then investigated whether the IVH-CSF could affect the differentiation of hfNPCs. The relative expression of *Gfap* mRNA in hfNPCs increased over 30 fold in cells treated with early IVH-CSF (early vs. ctrl adj-*p* = 0.0032) and over 15 fold in cells treated with late IVH-CSF (late vs. ctrl adj-*p* = 0.0338; early vs. late adj-*p* = 0.0128) compared to non-treated cells (Figure 4B). Exposure to both early (early mean:9.560, non-treated mean:1, SEM: 1.503, adj-*p* = 0.0006) and late IVH-CSF (late mean:7.235, non-treated mean: 1, SEM:1.503, adj-*p* = 0.0102) resulted in similar increase in the numbers of GFAP+ cells compared to non-treated cells (Figures 4A,G, Supplementary Figure 4). Exposure to both early (early mean: 236.2, non-treated mean: 132.3, SEM: 22.03, adj-*p* = 0.005) and late IVH-CSF (late mean: 221.9, non-treated mean:132.3, SEM: 22.03, adj-*p* = 0.0152) resulted in a similar increase of the cell area of GFAP^+^ cells (Figures 4A,H) compared to non-treated cells. Early but not late IVH-CSF resulted in increased GFAP protein expression (early mean:1.741, non-treated mean:1, SEM:0.1743, adj-*p* = 0.0112, Figures 4A,I) as well as the numbers of double GFAP+ and STAT3+ (early mean: 6.687, non-treated mean:1, SEM: 0.9930, adj-*p* = 0.0006, Figures 4A,C). Interestingly, the treatment with IVH-CSF had no effect on neuronal (Tuj1^+^ cells) or total cell numbers (Hoechst^+^ nuclei; Supplementary Figure 5). Together, these results suggest that IVH-CSF promotes a reactive astrocyte phenotype from neural stem pools.

To confirm that these effects on astrocytes were indeed mediated by JAK/STAT signaling, we repeated these experiments in the presence of a JAK inhibitor (JAK Inhibitor I). The number of GFAP+ and STAT3+ cells as well as levels of STAT3 phosphorylation were not affected by JAK Inhibitor (Figures 4A,D,F,G). However, JAK inhibition did reduce both GFAP expression levels (JAKinh(+) mean: 1.136, JAKinh(−) mean:1.741, SEM:0.1743, adj-*p* = 0.0416) and the cell area of GFAP+ cells (JAKinh (+) mean: 159, JAKinh(−) mean: 236.2, SEM: 22.03 adj-*p* = 0.0391, Figures 4A,H,I). Interestingly, JAK inhibition was only effective on cells treated with early IVH-CSF and GFAP levels remained elevated in cells treated with late IVH-CSF treated cells even after treatment with JAK inhibitor (JAKinh(+) mean: 7.186, JAKinh(−) mean: 1, SEM: 1.503, adj-*p* = 0.0108, Figures 4A,G,I). Furthermore, JAK/STAT inhibition caused an increase in MAPK phosphorylation in cells treated with late IVH-CSF but not early (JAKinh(+) mean: 1.096, JAKinh(−) mean: 0.6293, SEM: 0.1396,*p* = 0.0307, Supplementary Figure 3). These results show that the JAK/STAT pathway mediates the reactive gliosis seen at the early stages of IVH progression and that signaling through other signaling pathways – such as MAPK – becomes important at later stages of IVH progression.

## 4. Discussion

In the current study we aimed to characterize key signaling molecules – miRs – in the CSF of preterm infants with germinal matrix- intraventricular hemorrhage. miRs are differentially expressed in the CSF of premature infants over the course of GM-IVH. We performed next generation sequencing of the whole miR transcriptome in CSF samples from preterm infants following severe developing GM-IVH. We uncovered 587 miRs that are differentially expressed during progression of GM-IVH. GO analysis identified dysregulated pathways involved in ongoing brain development and the effects of the brain injury after the hemorrhage. By studying those dysregulated pathways we can begin to understand how this abnormal signaling might affect neural development in premature infants. Due to the limited access to human fetal and neonatal tissue, most previous findings have arisen from animal or adult studies. To our knowledge, this is the first study to explore the whole CSF-miR transcriptome in the developing human brain in the context of IVH, offering new insights into the miR involvement for both human brain development and disease.

Our gene ontology analysis revealed that the significantly dysregulated miRs are involved in cellular responses to insulin-like growth factor (IGF) stimulus. IGF is an important regulator of fetal brain development. Studies show that the levels of serum IGF-1 are decreased in preterm infants, while clinical trials have shown that supplementation with recombinant human (rh) IGF-1/rhIGF-binding protein 3 (rhIGF-1/rhIGFBP-3), prevents IVH (Ley et al., 2019). IGF-2, from CSF, was also found to support neural progenitor survival and proliferation (Lehtinen et al., 2011). Our GO analysis also points to metabolic dysregulation. Accordingly, Fame et al., showed that the metabolic shift from glycolysis to oxidative phosphorylation of central nervous system progenitor cells during very early development, can alter the composition of CSF (Fame et al., 2019). As such, the CSF composition may reflect the concurrent metabolic status of the adjacent brain.

Importantly, 310 miRs dysregulated with IVH were associated with the control of MAPK pathway, and 106 dysregulated miRs with the control of the JAK/STAT cascade. Furthermore, STAT3, one of the JAK/STAT pathway members, emerged as common target of 4 out of 9 dysregulated miR families. MAPK pathway plays an important role in cell proliferation, differentiation, and death (Morrison, 2012), while the JAK/STAT pathway is a well-studied pathway associated with reactive astrocytes (Haim et al., 2015; Ceyzériat et al., 2016, 2018). Astrocytes play a central role in central nervous system injuries and, depending on the time and context of their activation, can either aggravate (A1 astrocytes) or ameliorate (A2 astrocytes) tissue repair (Li et al., 2019). Distinct inflammatory stimuli can lead to activation of intracellular signaling molecules in astrocytes, with the MAPK, NFκB, and STAT3 activation among the main ones, while the timing of the activation and the crosstalk between those pathways are known to play a role on the astrocytic response (Colombo and Farina, 2016). For example, IL-6 activates the glycoprotein gp130 which can sequentially activate both STAT1/3 and MAPK (SHP2/Ras/ERK) cascades (Haroon et al., 2011). Interestingly although both pathways can control aspects of astrogliosis, Haroon et al. demonstrate that protective astrocyte activation is dependent on SHP2/Ras/ERK and not STAT3, at least in the context of experimental autoimmune encephalomyelitis. On the other hand, studies have shown that STAT3 activation induces SOCS3, a JAK/STAT inhibitor (in a negative loop), and that blockage of SOCS3 in astrocytes also enhances MAPK activation, showing the pleiotropic nature of some signaling molecules that well-studied regulators of one pathway may affect other signaling steps as well (Ma et al., 2010). Our results show that early IVH-CSF increased the STAT3+ differentiated human fetal NPCs, while late IVH-CSF decreased MAPK+ differentiated hfNPCs. These results suggest that these pathways are differentially and sequentially involved over IVH progression.

STAT3 has been associated with increased glial fibrillary acidic protein (GFAP) expression and reactive gliosis (Haim et al., 2015; Ceyzériat et al., 2016, 2018)and several studies have shown an increase in GFAP in the brain both in preclinical models of IVH (Deren et al., 2010) and in the CSF of preterm infants with GM-IVH (Whitelaw et al., 2001). Accordingly, our results show that IVH-CSF causes reactive gliosis in human fetal neural progenitor cells (increasing both GFAP intensity and GFAP+ cell number). This effect was specific to astrocytes as IVH-CSF did not change the percentage of Tuj1+ cells (neuronal marker) or the total cell number. We then showed that IVH-CSF-induced astrogliosis can be partly reverted with JAK inhibitor I. Of note, there are already FDA approved drugs targeting this pathway for some diseases [e.g., Rheumatoid Arthritis (Harrington et al., 2020)]. However, while STAT3-mediated reactive gliosis has been noticed in neonatal brains after injury, deletion of STAT3 in astrocytes exacerbated the white matter injury (Nobuta et al., 2012). Therefore, further investigation of JAK/STAT pathway on A1 “toxic” astrocytes specifically during the beginning of IVH might offer a viable and more targeted treatment for these infants.

Due to the key role of miRs in many biological processes and disease progression, miRs have been suggested as diagnostic/prognostic biomarkers in different diseases such as cancer (Wang et al., 2018) and neurodegenerative conditions (van den Berg et al., 2020). miRs have also been proposed as potential therapeutics while some are being assessed in interventional clinical trials (Hanna et al., 2019). Despite the lack of age-matched control CSF from healthy individuals (as there is no other ethically acceptable indication for performing invasive CSF sampling via lumbar puncture) our study illustrates how RNAseq from CSF (that would have otherwise been discarded), can identify miRs with common mRNA targets that together orchestrate key pathophysiological mechanisms. Further, *in vitro* and *in vivo* validation of our results will contribute to the development of potential interventions aimed at improving the neurodevelopmental outcomes in IVH infants.

## Data availability statement

The data presented in the study are deposited in the NCBI repository, accession number PRJNA1000302.

## Ethics statement

The studies involving humans were approved by the NHS Research Ethics Committee (REC number: 15-YH-0251). The studies were conducted in accordance with the local legislation and institutional requirements. Written informed consent for participation in this study was provided by the participants’ legal guardians/next of kin.

## Author contributions

AG: experimental design, data acquisition, analysis and interpretation, and manuscript preparation. RS: hfNPC data acquisition and analysis. JU and TP: provided expertise and intellectual input. OCL and AH: project leads, experimental design, data interpretation, and manuscript preparation. AG, RS, TP, JU, OCL, and AH: critical review of final manuscript. All authors contributed to the article and approved the submitted version.

## Funding

This work was funded by the University of Oldenburg, Germany. This work was also supported by Charity SPARKS & Castang Foundation.

## Conflict of interest

The authors declare that the research was conducted in the absence of any commercial or financial relationships that could be construed as a potential conflict of interest.

## Publisher’s note

All claims expressed in this article are solely those of the authors and do not necessarily represent those of their affiliated organizations, or those of the publisher, the editors and the reviewers. Any product that may be evaluated in this article, or claim that may be made by its manufacturer, is not guaranteed or endorsed by the publisher.

## Footnotes

^1^https://www.htgmolecular.com/assays/mirna-wta

^2^https://www.htgmolecular.com/reveal
