# Supplementary material for: The miRNA transcriptome of cerebrospinal fluid in preterm infants reveals the signaling pathways that promote reactive gliosis following cerebral hemorrhage
Source: Front Mol Neurosci. 2023 Sep 18;16:1211373. doi: 10.3389/fnmol.2023.1211373 (PMC10544345; doi:10.3389/fnmol.2023.1211373)
Supplement: Supplementary file 1 [file Data_Sheet_1.docx]

***Supplementary Material***

**The miRNA transcriptome of cerebrospinal fluid in preterm infants reveals the signalling pathways that promote reactive gliosis following cerebral haemorrhage**

**Andriana Gialeli^1^, Robert Spaull^2^, Torsten Plösch^1^, James Uney^2^, Oscar Cordero Llana^2,#^, Axel Heep^1,#^**

*** Correspondence:** Axel Heep, email: axel.heep@uni-oldenburg.de

**Supplementary Figures and Tables**

**Supplementary Figures**


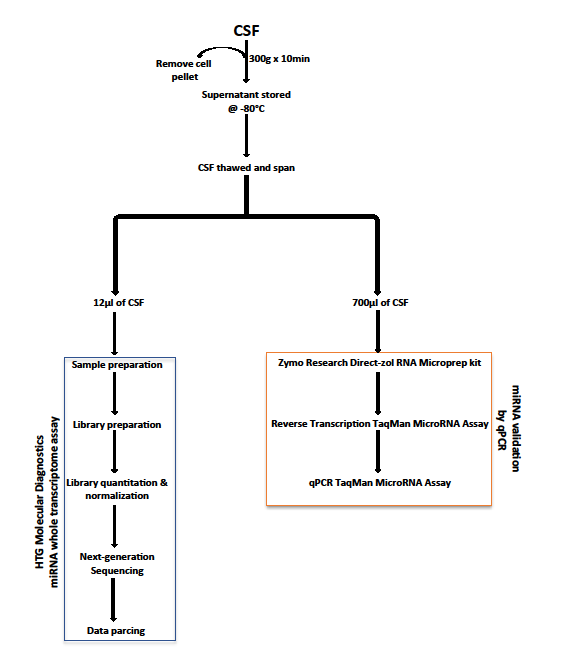


**Supplementary Figure 1.** Workflow of the experimental process for miRNA expression profiling.


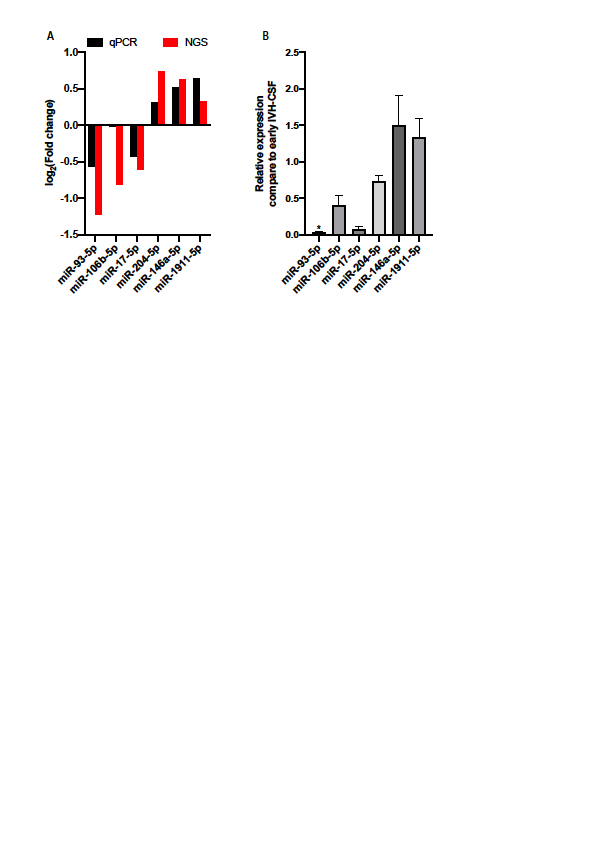


**Supplementary Figure 2.** A) Comparison of direction of expression fold change between qPCR and NGS of 6 deregulated miRa. B) Relative expression of 6 deregulated miRs in late IVH-CSF compared to early IVH-CSF, tested by qPCR (n=5 independent CSF samples, Dunnett’s multiple comparison Test *p=0.0193). Bars represent mean+SEM.


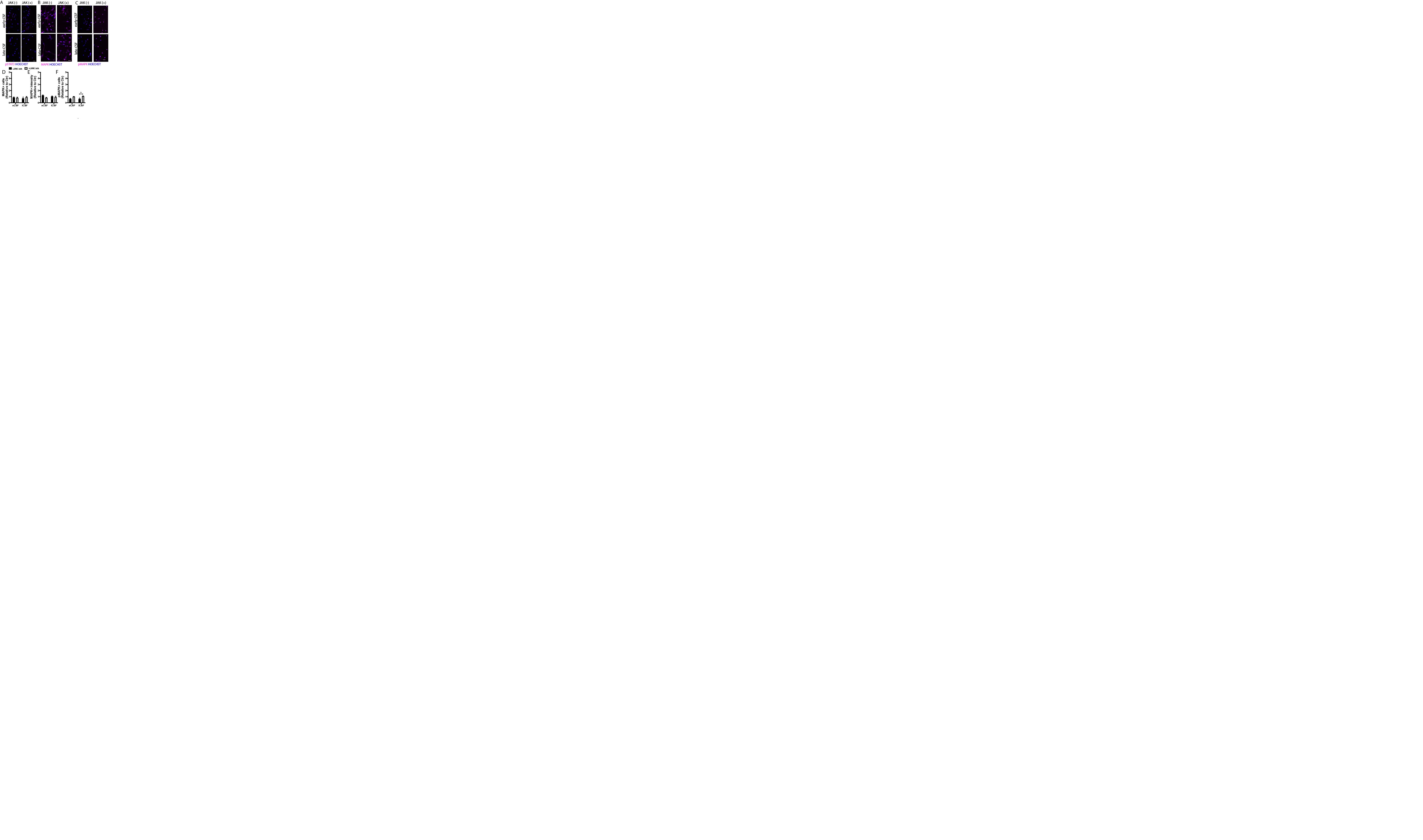


**Supplementary Figure 3.** (A-C) Representative immunofluorescence images of human foetal NPCs treated with either early- or late-CSF with/without JAK inhibitor stained for pSTAT3, MAPK and pMAPK. Scale bars =50μm. (D) Relative quantification of the number of MAPK^+^ cells. (E) Relative quantification of MAPK intensity. (F) Relative quantification of the number of phosphorMAPK^+^ cells. All experiments were repeated with n=3 independent CSF samples. (*) represents the significant difference compared to non-treated cells, (δ) represents the significant difference between JAKinh (+) and JAKinh (-).


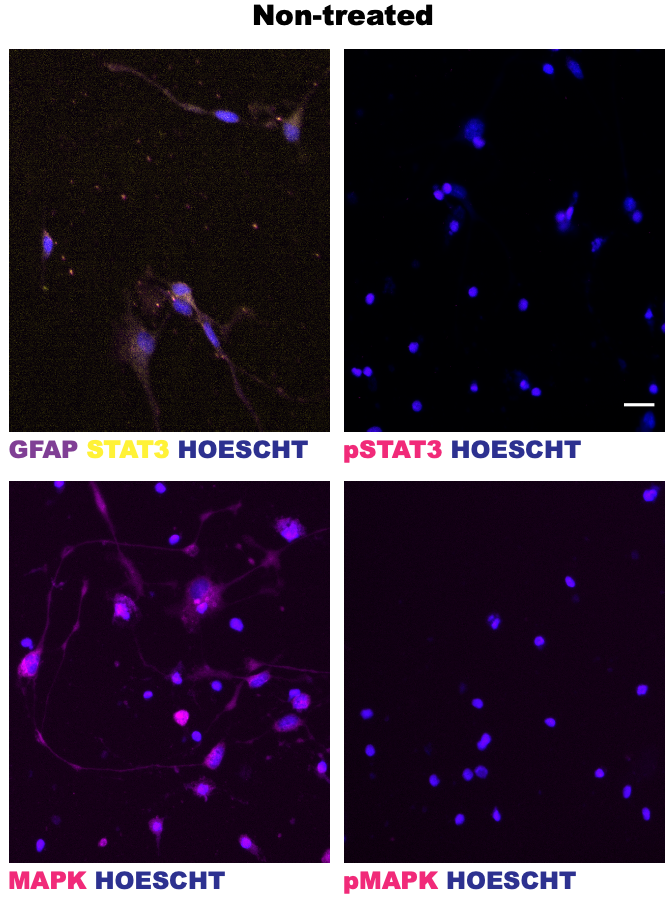


**Supplementary Figure 4.** Representative immunofluorescence images of non-treated human foetal NPCs stained for GFAP, STAT3, pSTAT3, MAPK, pMAPK. Scale bar 50μm.


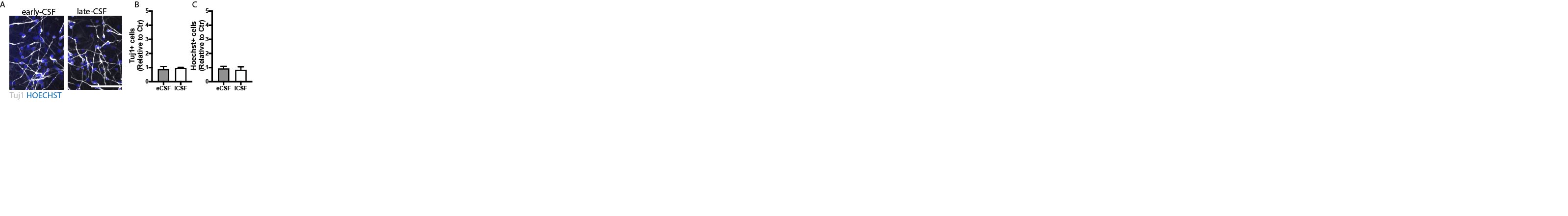


**Supplementary Figure 5.** (A) Representative immunofluorescence images of human foetal NPCs treated with either early- or late-CSF stained for Tuj1. Scale bar 50μm. (B) Relative quantification of the number of Tuj1+ cells. (C) Relative quantification of the number of Hoechst+ cells. All experiments were repeated with n=3 independent CSF samples.

**Supplementary Tables**

**Supplementary Table 1.** Patients’ information.

| **Patient** | **Sex** | **Gestational age at birth** | **Age of samples (days)** | **Weight at birth (kg)** | **IVH grade*** |
| --- | --- | --- | --- | --- | --- |
| **1** | Female | 37 weeks | 4 | 2.8 | No** |
| **2** | Male | 24 weeks | 8 | 0.7 | III |
| **3** | Male | 24 weeks | 20 | 0.7 | IV |
|  |  |  | 121 |  |  |
| **4** | Male | 24 weeks | 20 | 0.7 | IV |
|  |  |  | 68 |  |  |
| **5** | Male | 24 weeks | 21 | 0.7 | III |
| **6** | Male | 24 weeks | 29 | 0.6 | III |
|  |  |  | 134 |  |  |
| **7** | Female | 24 weeks | 29 | 0.6 | IV |
|  |  |  | 88 |  |  |
| **8** | Female | 23 weeks | 30 | 0.6 | III |
|  |  |  | 84 |  |  |
| **9** | Female | 26 weeks | 32 | 0.7 | III |
| **10** | Female | 24 weeks | 33 | 0.5 | III |
| *maximum IVH grade recorded from either right or left ventricle, ** Congenital Ventriculomegaly | | | | | |

**Supplementary Table 2.** Antibodies’ epitopes, catalogue numbers and suppliers.

| **Antibody**  **Target** | **Epitope** | **Supplier** | **Catalogue**  **Number** | **Dilution** |
| --- | --- | --- | --- | --- |
| **GFAP** | GFAP isolated from cow spinal cord. | DAKO | Z0334 | 1:1000 |
| **Tuj1** | Microtubules derived from rat brain. | Biolegend | 801202 | 1:500 |
| **STAT3** | Synthetic peptide centred around amino acid Gin692 of human Stat3. | Cell Signalling | 9139T | 1:200 |
| **pSTAT3**  **(Tyr705)** | Synthetic phosphopeptide corresponding to residues surrounding Tyr705 of mouse Stat3. | Cell Signalling | 9145T | 1:200 |
| **MAPK** | Synthetic peptide corresponding to a sequence in the C-terminus of rat p44 MAP Kinase. | Cell Signalling | 9102 | 1:200 |
| **pMAPK (Thr202/Tyr204)** | Synthetic phosphopeptide corresponding to residues surrounding Th202/Tyr204 of human p44 MAP Kinase. | Cell Signalling | 4377B | 1:200 |

**Supplementary Table 3.** Primers’ sequences, IDs and suppliers

| **miR-base ID** | **Mature miRNA Sequence** | **Expression** | **TaqMan MicroRNA Assay ID,**  **(Cat #: 4427975, Applied Biosystems^TM^, ThermoFisher)** |
| --- | --- | --- | --- |
| **hsa-miR-146a-5p** | UGAGAACUGAAUUCCAUGGGUU | Top-upregulated based on our NGS analysis | 000468 |
| **hsa-miR-204-5p** | UUCCCUUUGUCAUCCUAUGCCU | Top-upregulated based on our NGS analysis | 000508 |
| **hsa-miR-93** | AAAGUGCUGUUCGUGCAGGUAG | Top-downregulated based on our NGS analysis | 000432 |
| **hsa-miR-106b-5p** | UAAAGUGCUGACAGUGCAGAU | Top-upregulated based on our NGS analysis | 000442 |
| **hsa-miR-17-5p** | CAAAGUGCUUACAGUGCAGGUAGU | Previously reported to be downregulated in exosomes isolated from GM-IVH CSF (Spaull *et al.,* 2019) | 000393 |
| **hsa-miR-1911-5p** | UGAGUACCGCCAUGUCUGUUGGG | Previously reported to be upregulated in exosomes isolated from GM-IVH CSF (Spaull *et al.,* 2019) | 121116_mat |
| **cel-miR-39-3p** | UCACCGGGUGUAAAUCAGCUUG |  | 000200 |
| **Target** | **Primer Sequence** | **Source** |  |
| **Gfap** | Forward: AGATCCACGAGGAGGAGGTT  Reverse: ATACTGCGTGCGGATCTCT | Merk |  |
| **Stat3** | Forward: GCCTCTGCCGGAGAAACA  Reverse: TCCAGGTACCGTGTGTCAAG | Merk |  |
| **Gapdh** | Forward: TGCACCACCAACTGCTTAGC  Reverse: GGCATGGACTGTGGTCATGAG | Merk |  |

**Supplementary Table 4.** The table shows the family members of the deregulated miR families and their seed sequence.

|  |  |  |
| --- | --- | --- |
| Family Name | **Family Member** | **Seed Sequence** |
| miR-548 | miR-548d-5p/miR-548ay-5p/miR-548w/miR-548b-5p/miR-548ak | AAAGUA |
| miR-17 | miR-106b-5p/miR-93-5p/miR-17-5p/miR-3609 | AAAGUG |
| miR-3689 | miR-3689a-3p/miR-6779-5p/miR-3689b-3p/miR-1273h-5p | UGGGAG |
| miR-378 | miR-378h/miR-378e/miR-378f | CUGGAC |
| miR-6870 | miR-6870-5p/miR-5698/miR-5010-5p/miR-7111-5p/miR-625-5p | GGGGGA |
| miR-320 | miR-320c/miR-320d/miR-4429 | AAAGCU |
| miR-30 | miR-30d-5p/miR-30e-5p/miR-30a-3p | GUAAAC |
| miR-4251 | miR-4251/miR-6761-5p/miR-4329 | CUGAGA |
| miR-23 | miR-23c/miR-23b-3p/miR-23a-3p | UCACAU |
